# Household catastrophic health expenditure: evidence from Georgia and its policy implications

**DOI:** 10.1186/1472-6963-9-69

**Published:** 2009-04-28

**Authors:** George Gotsadze, Akaki Zoidze, Natia Rukhadze

**Affiliations:** 1 Curatio International Foundation (CIF), 37d. I Chavchavadze ave, Tbilisi, 0179, Georgia; 2 Curatio International Consulting (CIC), 37d. I Chavchavadze ave, Tbilisi, 0179, Georgia

## Abstract

**Background:**

To quantify extent of catastrophic household health expenditures, determine factors influencing it and estimate Fairness in Financial Contribution (FFC) index in Georgia to establish the baseline for expected reforms and contribute to the design and fine-tuning of the major reforms in health care financing initiated by the government mid-2007.

**Methods:**

The research is based on the nationally representative *Health Care Utilization and Expenditure *survey conducted during May-June 2007, prior to preparing for new phase of implementation for the health care financing reforms. Households' catastrophic health expenditures were estimated according to the methodology proposed by WHO – Ke Xu [1]. A logistic regression (logit) model was used to predict probability of catastrophic health expenditure occurrence.

**Results:**

In Georgia between 2000 and 2007 access to care for poor has improved slightly and the share of households facing catastrophic health expenditures have seemingly increased from 2.8% in 1999 to 11.7% in 2007. However, this variance may be associated with the methodological differences of the respective surveys from which the analysis were derived. The high level of the catastrophic health expenditure may be associated with the low share of prepayment in national health expenditure, adequate availability of services and a high level of poverty in the country. Major factors determining the financial catastrophe related to ill health were hospitalization, household members with chronic illness and poverty status of the household. The FFC for Georgia appears to have improved since 2004.

**Conclusion:**

Reducing the prevalence of catastrophic health expenditure is a policy objective of the government, which can be achieved by focusing on increased financial protection offered to poor and expanding government financed benefits for poor and chronically ill by including and expanding inpatient coverage and adding drug benefits. This policy recommendation may also be relevant for other Low and Middle Income countries with similar levels of out of pocket payments and catastrophic health expenditures.

## Background

Georgia is a lower-middle-income country, according to the World Bank classification with Gross National Income (GNI) per capita $1,560 in 2006 [2]. After gaining independence from Soviet Union in 1991, Georgia faced the deepest economic shock among all former Soviet republics. Between 1990–1995 economic output declined by 78% [3], which brought annual public expenditure on health down to 80 cents (US) in per capita terms. In response to the declining public spending during 1996–1997, the government of Georgia, as other countries of former socialist block in Europe and Central Asia, has embarked on major health sector reforms, which separated health care provision from financing, helped the country establish a single purchaser in 1999 that contracted providers and introduced output-based payments as the predominant form of provider reimbursement. Structural reforms allowed the government to remove up to 180,000 health care workers from the state payroll and devolved hiring and firing powers onto autonomous (but publicly owned) health care facilities, which emerged as a result of these reforms. In light of limited public spending on health and a very narrow benefit package, private out-of-pocket payments emerged as a predominant source of financing service provision. Most of personal health care services, as in many of low and middle income countries, were paid on a fee-for-service basis by the population [4]. According to various estimates the share of out-of-pocket payments (OOP, both formal and informal) in *Total Health Expenditure *(THE) reached 80% [5,6] in 2002. But since, growing public spending for health, increasing along with economic growth observed in the country during recent years, allowed decreasing slightly the share of private expenditure in THE. However, according to a recent national health accounts exercise, this share still stands at high level of 72% of THE [7] for 2006. This is the highest level of private expenditures on health not only in the European Region (app. 25% in average), but also exceeds the CIS average (app. 46%) [5].

The growing OOP spending in the health sector became the significant factor contributing to impoverishment of Georgian households [8] and attracted the government's attention. Since 2001, the government created a separate publicly funded national program that has offered increased health care benefits to poor. However, the administrative system used to deliver subsidies to poor was inherited from the Soviet Union and was based on social categorical groups (e.g. internally displaced, war veterans, etc.). This system significantly limited actual impact of the state health subsidies for poor. In 2004, the government started developing a proxy-means-tested system for the detection of poor households and delivery of the state subsidies (cash and in-kind). Mid 2006, this new administrative system became functional throughout the country and allowed for delivering targeted health care benefits to poor households in addition to poverty cash benefits.

Subsidized health care benefits for poor were converted into entitlement vouchers that were distributed to all the eligible poor with the help of new social assistance system. These benefits include a comprehensive package of outpatient services and coverage for emergency hospital care. Limited elective hospitalizations are allowed, but are rationed through waiting lists (up to two-three month). Outpatient prescription drugs are considered for inclusion, although yet they are not part of the state funded benefits for the poor. The total budget for this program is capped with the strict budgetary constraint, not allowing arrears to accrue and assuring the financial stability of the system.

While the extent of out-of-pocket expenditure on health is well known, the evidence describing whether it is catastrophic or not is lacking. By looking at utilization and expenditure for medical care incurred by various population groups a year later after initiation of the state funded program for the poor, this paper attempts to evaluate the prevalence of catastrophic health expenditure and its determining factors. It also estimates the Fairness in Financial Contribution (FFC) index to establish the baseline prior to next phase of reforms planned for 2008, when the government intends to extend coverage with targeted health benefits from current 660,000 beneficiaries to 1,200,000 individuals that considered to be poor (35.5% of total population) and also contract out the delivery of these benefits to private insurance companies. It is yet debated how this major shift to "commercializing" health sector in Georgia would affect the financial protection of poor and effectiveness of public policy aimed at delivering health care benefits to those the most in need.

## Methods

We used the nationally representative *Health Utilization and Expenditure Survey *(HUES) implemented by the National Statistical Office during May-June 2007 [9]. This was the focused survey aimed at estimating household health care utilization and expenditure and providing a baseline for reforms planned in the primary health care and health care financing. The households in this survey, in its design were linked with the *Integrated Household Survey *(IHS) implemented on a quarterly basis by the National Statistical Office. This link was established for the purposes of merging household characteristics (economic, social, etc.) with the healthcare utilization and expenditure of the given household. Total 2,859 households were associated/linked in these two surveys and our analysis is based on the findings from these households.

### Structure of the HUES Questionnaire

The questionnaire was developed by drawing on a number of existing questionnaires that had already been used in Georgia before and consisted of seven sections: a) household composition and demography; b) self-reported health status of household members; c) availability of health care facilities to the household; d) last medical service used by any household member during last 6 months, which provided information for each household member who had a medical consultation (including preventive service) in the last six month. This section primarily helped us evaluate service utilization (not expenditures); e) services used and associated costs for illnesses that occurred during 30 days prior to the interview. This information was collected for each person who has been sick and used health services or spent any money on health care in the last 30 days. This section primarily helped estimate health care expenditures for outpatient, diagnostic and drug services; f) hospitalizations during the last year completed for anyone who has been hospitalized within the last one year but not in the last 30 days (because these individuals were captured in the previous section of the survey tool); g) occasions when individuals were not hospitalized but should have been.

### Type of expenditures used in HUES

The survey instrument allowed for looking at following average monthly expenditures (on a household level): inpatient, outpatient, recurrent costs for chronic conditions. Each group of expenditure was also partitioned into expenditures for medications, medical supplies, diagnostic and consultation fees, and nursing and physiotherapy.

### Catastrophic Health Expenditure

The concept of catastrophic health expenditure has been defined as occurring once out of pocket payments cross estimated threshold share of household expenditure at which the household is forced to sacrifice other basic needs, sell assets, incur debt or be impoverished [10-12]. Thresholds used by different researchers to estimate catastrophic health payments vary from 5 percent to 20 percent of total income, or 12.5–50 percent of non-subsistence income for poor families spending 60 percent of their income on food [13]. While there is no final consensus on the choice of the threshold, for this paper we employ more frequently used threshold proposed by the researchers at World Health Organization in their "fair financing" framework [14]. K. Xu et al define catastrophic health expenditure in relation to the households' nonfood expenditures. The health expenditure is determined as being catastrophic if a household's financial contributions to the health equals and/or exceed 40% of nonfood expenditure or *Capacity to Pay *(CTP). A CTP was estimated after subtracting *Subsistence Expenditure *from monthly household expenditure (i.e. consumption) obtained from the HIS survey. *Subsistence Expenditure *for the purposes of our calculations corresponds to the average food expenditure of the households in the 45^th ^and 55^th ^percentile, adjusted to the size of the given household [15]. To adjust for household size we used *Consumption Equivalence Scale *and the methodology suggested by K. Xu et al. 2003 [15]. To compare households with different economic status, expenditure quintile groups were defined through ranking household monthly expenditure per adult equivalent (dividing household monthly expenditure by adult equivalent household size).

### Fairness in Financial Contribution

Fairness in Financial Contribution (FFC) is defined by WHO to be one of the three intrinsic goals of a health system. The FFC index measures whether a country collects contributions from households to finance health in an equitable manner [16]. It captures the extent of catastrophic health spending by households. Therefore, FFC index was used to assess the distribution of household financial contribution. This index weighs heavily those households that have spent a very large share of their beyond subsistence resources on health. The index thus reflects overall inequality in household financial contribution into the health system, but particularly reflects those households facing catastrophic health expenditure. The FFC index was calculated based on methodology suggested by WHO [17]. The FFC index is based on the mean of the cubed absolute difference between the **out-of-pocket health payments share of household capacity to pay (oopctp)** in a given household and the norm of the same indicator. The index is of the form:



The FFC index ranges between 0 and 1. The fairer the health financing system, the closer FFC index will be to 1.

### Statistical Analysis

SPSS™ Software was used for statistical analysis. A descriptive analysis was undertaken to understand occurrence of illness, care seeking behavior and size of out-of-pocket payments on a household level. A logistic regression (logit) model was used to predict probability of catastrophic health expenditure occurrence. Based on evidence available elsewhere in the literature, we assumed that households having catastrophic expenditure are affected by patterns of illness and type/place of treatment they receive (facing expenditure due to chronic illness, facing cost of hospitalizations and receiving care in the capital city or in the regions, where cost of treatment could be less due to lower level of care and type of services available), household characteristics which includes household size, their vulnerability status (eligibility to state subsidies), education of the head of household and finally HH's economic status (measured by quintile group) [8,13,18,19]. All these variables were entered in the Logit model using forward stepwise entry function in the SPSS software. Variable was included in the model if the probability of its score statistic was less than 0.05 and was removed if the probability was greater than 0.1. The stepwise entry-removal of the various explanatory variables allowed identifying those that had statistically significant influence on the probability of determining catastrophic health expenditure. These variables were: a) households with expenditure for treating chronic illness and b) households that faced hospital expenditure (both included in the model as dichotomous variable); c) quintile groups and d) geographical location of the household included in the model as categorical variables. All households in the sample were distributed among three conventional geographical regions: the capital city of Tbilisi, which houses third of the country's population and almost all tertiary care facilities; and East and West Georgia, which are mainly rural areas with small number of urban locations. The probability of catastrophic health expenditure was calculated with the Logit model [20] and the model goodness-of-fit was assessed by Hosmer-Lemeshow test [21].

## Results

### Morbidity

A total of 10,445 individuals resided in the surveyed 2,859 households and the mean household size was 3.65 (SD 1.9). The survey asked respondents to distinguish between chronic and acute illnesses, with the former being defined as ones that had lasted or were expected to last more than one year. Correspondingly, the results for the two were reported separately. The proportion of people, who reported suffering from a chronic illness, was high – 37%, with 11% of the population reporting suffering from two or more chronic illnesses. There was a wide range of chronic conditions reported but the most common chronic diseases were hypertension and other heart or circulatory diseases, which accounted for about a third of all occurrences. Some 15.6% of the respondents reported having had an acute sickness during the last thirty days and 9% had both an acute sickness and a chronic illness (see Table 1). Most of the acute sicknesses were respiratory diseases (42% of all occurrences) and cardiovascular diseases. Overall, around half (51%) of the population rated their health as good, or better than good, over the last four weeks. The rural population was slightly less likely to say this, but differences were small and statistically not significant.

**Table 1:** Key indicators by consumption quintile (individual level data)

**Indicator**	**poorest** **fifth**	**2**	**3**	**4**	**richest** **fifth**	**Total %**	**N**
% of total population with chronic disease	34.1	37.0	37.3	38.0	38.6	37.0	10,445
% of population with chronic disease and consulting healthcare provider	52.3	55.4	56.5	59.7	64.4	57.7	3,862
% of total population with acute sickness during last 30 days	14.3	14.9	16.7	15.0	17.5	15.6	10,445
% of those sick during last 30 days and consulting healthcare provider	63.4	63.3	66.0	62.7	63.5	63.8	1,634
% of patients who were able to obtain medications prescribed by doctor during last consultation	79.1	83.8	85.2	83.0	90.1	84.3	4,946
% of consultations where medicine was prescribed but not purchased because it was too expensive (base: all consultations)	16.4	11.6	11.6	12.2	7.3	11.8	4,946
% reported to be beneficiaries of State Program for Population below the Poverty Line	20.3	17.1	12.1	12.7	6.5	13.6	5,496

*Richer households were slightly more likely to report illness than poor, although this probably reflects different perceptions of illness. This is consistent with *other studies elsewhere showing that the proportion of self-reported illness is less significant among the poor than the non-poor. Even though the poor might suffer more illness than the non-poor, the non-poor perceive themselves to suffer as much and to have even more illness than the poor [22].

### Health service utilization

*Richer households were also more likely to consult a health care provider when they are sick. The differentials in utilization between income groups were not large and depended somewhat on which measure was used – there are no appreciable differences in the proportion consulting a healthcare provider when individuals with an acute illness were analyzed alone *(see Table 1).

The survey also looked at physical access measures – distance and availability of services. In terms of physical access, most people had access to a health facility within 30 minutes by their usual means of transport (bus or walking). Even in rural areas, 81.5% of the population lives within 30 minutes of the health facility that is the nearest and/or normally visited (92.8% in urban areas and 72.2% in rural). However, small fraction of the population has to travel longer distances.

### Healthcare spending

The survey captured monthly healthcare expenditure that was broken down by several categories: a) recurrent costs (mainly cost of drugs and some medical items) faced by households due to chronic conditions; b) cost of outpatient care when healthcare provider was consulted; c) costs of self-treatment without consulting health care provider. Mean costs in all three categories were lowest among the poorest quintile groups and highest among the richest (see Table 2). In addition, the survey only captured 45 cases of hospital utilization during 30-day period prior to interview and the cost of hospitalization ranged from 2000 to 5 Gel per case of hospital admission.

**Table 2:** Household monthly expenditure characteristics *mean and (95% CI)*

**Quintile** **Groups**	**N**	**Monthly Household** **Expenditure (Gel)^1^**	**Monthly Recurrent** **Costs for Chronic** **Conditions (Gel)**	**Total outpatient care** **costs (Gel)**	**Total self-treatment** **costs (Gel)**
Poorest	604	125.9 (125.7:126.2)	7.3 (7.3:7.4)	3.1 (3.1:3.1)	1.7 (1.7:1.7)
2	573	232.3 (232:232.6)	12.8 (12.7:12.9)	8.1 (8:8.2)	2.0 (2.0:2.1)
3	566	328.0 (327.5:328.4)	17.7 (17.6:17.8)	11.1 (11:11.3)	4.5 (4.4:4.5)
4	572	457.7 (457.1:458.3)	22.1 (21.9:22.2)	18.6 (18.4:18.8)	3.4 (3.4:3.5)
Richest	544	821.7 (819.9:823.5)	25.0 (24.8:25.2)	49.4 (48.6:50.2)	6.7 (6.6:6.8)

**Total**	**2859**	**393.2 (392.5:393.8)**	**17 (16.9:17.1)**	**18.1 (17.9:18.2)**	**3.7 (3.6:3.7)**

The survey findings also indicate possible problems in targeting, despite the fact that survey was not designed specifically for benefit incidence analysis. Table 1 shows that while more than 20% of households from lowest income quintile are beneficiaries of the poverty health benefit, at the same time 6 to 12% of "rich" households representing the highest income quintiles are also recipients of the same program. This finding should be interpreted with caution, as eligibility for poverty health benefit is determined using the "means testing" system, which implies more comprehensive assessment of a household economic status using up to 35 indicators and variables, while the IHS mainly assess the cash expenditures incurred by a household in a given time period. Further inquiry in this issue is warranted, but this issue is beyond the focus of this paper.

### Catastrophic expenditures on health

**Figure 1:**
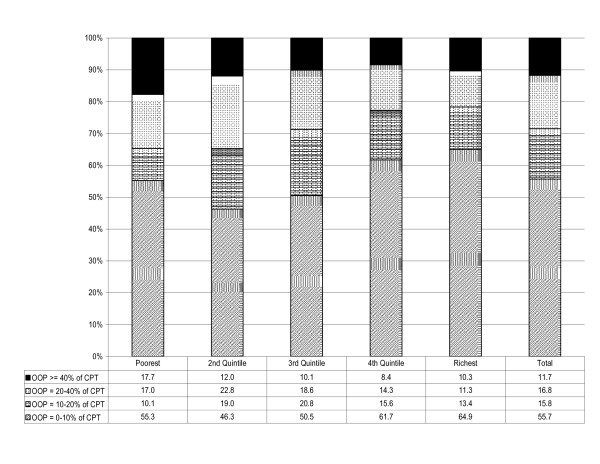
**% of households (by quintile groups) facing different levels of health expenditure at different cut-off points**.

In our sample, the households that faced catastrophic health care expenditure amounted to 11.7% and the poorest quintile had the highest share – 17.7% (see Figure 1). In addition, residents of the capital city were more likely to face catastrophic health care costs 14.8% than residents of East and West Georgia (11.2% and 10.1% respectively). This possibly could be due to higher costs of more complex health care services available in the capital and relatively easy access to facilities in the capital city.

### Determinants of catastrophic health expenditures

Logistic regression revealed (see Table 3) that the odds of facing catastrophic expenditure were 4.4. and 27 times higher among households having incurred expenditure for treating chronically ill persons and those that had case of hospitalization. Households in the richest quintile were four times less likely to face catastrophic expenditure when compared with the poorest quintile and as the households' monthly consumption increased probability of facing catastrophic health expenditure declined. Finally, the odds of facing catastrophic health spending were almost two times higher for the capital city residents compared to those households that received care in East and West Georgia.

**Table 3:** Estimated coefficients in Logit model for catastrophic health care expenditure (Household level data)

**Variable**	**B**	**Wald**	**P Value**	**Odds** **Ratio**	**CI 95.0% for** **Odds Ratio**
Intercept	-0.67	14.6	<0.001	**0.17**	
Chronic costs (1 if HH had recurrent cost due to chronic condition)	1.49	109.4	<0.001	**4.41**	(3.34: 5.83)
Hospitalization (1 if HH faced cost of hospitalization otherwise 0)	3.30	85.4	<0.001	**27.13**	(13.47: 54.64)
Poorest Quintile (Reference Group)		72.8	<0.001		
2nd Quintile	-0.82	20.7	<0.001	**0.44**	(0.31: 0.63)
3rd Quintile	-1.15	36.5	<0.001	**0.32**	(0.22: 0.46)
4th Quintile	-1.34	45.1	<0.001	**0.26**	(0.18: 0.39)
Richest Quintile	-1.30	41.3	<0.001	**0.27**	(0.18: 0.41)
Capital city Tbilisi (Reference group)		13.4	0.001		
East Georgia	-0.53	9.9	0.002	**0.59**	(0.42: 0.82)
West Georgia	-0.61	12.1	0.001	**0.55**	(0.39: 0.77)
					
Log likelihood	1,764.1				
Pseudo R^2^	0.085				
Hosmer-Lemeshow test	χ^2^(8) = 11.16				
	P = 0.19				
Observations	2,859				

^1 ^In May-June 2007, 1 USD was app. equal to 1.68 GEL (the exchange rate varied from 1.67 – 1.69 during this period)

Computation of FFC index for Georgia rendered 0.82, which shows that Georgia has relatively fair health care financing system, when compared to peer group of countries.

## Study limitations

The presented study faced the following limitations:

- The survey captured only the direct cost of care to the patient/household. The survey tool did not account for (a) the portion of the cost of services when paid by third party payers (insurance companies, government programs, etc) and (b) expenditures for paying state taxes and levies, certain portion of which commonly goes for financing health care services; Therefore the full impact of government subsidies and spending on the household level and its impact on the prevalence of catastrophic health spending can not be determined.

- Household expenditures estimated in this paper are those mainly made in cash (NB in-kind payments in Georgia's health care system are rare and therefore could be ignored) and the cost of transportation and economic costs to the households are not taken into account.

- Finally, all diseases and complaints are self-reported and while they may sometimes be based on diagnoses given by doctors to the respondents, in other cases they may not be.

## Discussion

Catastrophic health expenditure could only be measured when health services are used and costs of service provision paid. Many poor households simply avoid seeking care due to financial considerations, therefore presented figures could underestimate the reality. P. Saksena et al [23] also acknowledged this limitation and proposed broader framework for catastrophic health expenditure. They estimated the total potential (unobserved and observed) incidence of catastrophic health expenditure in Kenya by combining the reported out-of-pocket expenditures for those who utilized health services with the predicted out-of-pocket expenditures for those who did not use health services but reported illness. The authors found a significant difference between the total number of households potentially facing catastrophic expenditure and the households who actually faced catastrophic expenditure. This difference was more profound for households from poorest quintile – three times as many households would have faced the catastrophic expenditures in case of use of health services (19 percent vs.6.6 percent). While the risk of the catastrophic health expenditures for households in richest quintile who did not use services, was minimal [23]. Our analysis has shown that around 40% of the population, when sick with chronic or acute conditions, do not seek care from a medical provider. Therefore, the measure of the catastrophic health spending presented in this paper may well underestimate the real prevalence among Georgian population and most importantly among the poor. However, these findings deserve cautious interpretation, because several factors could affect such behaviour. One is the perception of seriousness of the illness. Past research in Georgia showed that those that perceive illness not to be serious are least likely to seek care [24]. In addition, patients with chronic conditions may choose to self-treat (using already prescribed medicine), because drugs (both prescription and non-prescription) could be easily purchased in Georgia and cost of self-treatment is usually lower then visiting the doctor [25]. We compared changes in utilization over time and used chronic conditions (as a tracer) to relate findings of HIS [26] from 2000 with ours. The comparison presented in Table 4 reveals that reporting of chronic illness has increased during this period, which could be a natural process or due to different methodologies used in these two different surveys. Notwithstanding these limitations, it becomes obvious that most of the increase in care utilisation over the period during 2000–2007 benefited mainly the poorest groups, because the gradient between the poorest and richest quintiles decreased from 18.3% in 2000 down to 11.7% in 2007. This finding is accentuated by the fact, that relative and absolute poverty rates had not changed considerably during this period (e.g. relative poverty level was 24% in 2000 and 21.3% in 2007) [27]. Respectively, the change in this gradient could be an indication that government's efforts to deliver better health coverage to poor have rendered some positive results. However, a direct causal link between government implemented policies and observed outcomes cannot be established by our study.

**Table 4:** Comparison between 2000 and 2007 surveys – Incidence and treatment of chronic illnesses (individual level)

	**IHS 2000**		**2007 HUES**	
	
Quintile	% of pop reporting sick	% of sick seeking care	% of pop reporting sick	% of sick seeking care
Poorest	12.2	42.9	34.1	52.3
2	11.8	49.5	37.0	55.4
3	11.8	51.1	37.3	56.5
4	12.5	61.1	38.0	59.7
Richest	13.3	61.2	38.6	64.0

Total	12.3	*53.3*	37.0	57.7

While during 2000–2007 access to care for the poor has improved slightly, the share of households that face catastrophic health expenditure have seemingly increased. In 2007 the share of households incurring catastrophic health expenditure reported by Xu et all [15], based on the analysis of the Household Budget Survey 1999, was 2.8% which was close to the mean figure for 89 countries analyzed by the authors. Our estimate of 11.7% of population based on the 2007 *Health Utilization and Expenditure Survey *puts Georgia on the top of the list – as having one of the most unprotected health care financing systems, along with other transition countries (Azerbaijan, Ukraine, Vietnam and Cambodia) that feature a similarly high rate. However, we think such international comparisons bear inherent limitations. Our study primarily focused on questioning health care utilization and expenditure, while most surveys used in the papers were either *Living Standard Measurement Studies*, or household budget surveys or household income and expenditure surveys that did not specifically look at health care utilization and expenditure. Consequently, a recall bias in non-health care surveys may underestimate spending levels on health, while our survey focused on health, possibly rendered higher estimates. The same situation is observed in other countries, e.g. in Azerbaijan the household budget surveys in 1995 and 2006 showed almost three times lower health expenditures than specially designed health utilization and expenditure surveys [28]. It was also the case when we compared HUES health expenditure estimates with HIS from 2007 [9]. Nevertheless, the share of household that face catastrophic health spending is high in Georgia and calls for policy solutions. Consequently, monitoring the rate over time, while using the same HUES survey tool, will allow the Government to observe changes in the future if they occur. Finally, Georgia has improved its FFC index, which was estimated at 0.68 in 2004 [29] and according to our survey findings stands at 0.82 for 2007. This figure will also serve as a baseline to assess the impact of the planned health sector reforms in future.

Overall, the survey findings are consistent with the research results in the literature – high level of catastrophic health expenditures in Georgia are probably predetermined by low level of prepayment (no more than one third of THE), good physical access to health services (as in many post Soviet countries) and high level of poverty (more than one fifth of the nation is considered to be living in poverty) [15].

### Policy Implications

Findings of the paper offer some suggestions for evidence-based policy solutions that will help decrease the prevalence of catastrophic health spending in Georgia. The Georgian government's drive for alleviating poverty and increasing incomes for poor households in medium to longer term perspective is expected to have a positive impact not only for health, but beyond. In the interim, health sector specific policies could focus on two target areas: a) increasing size of the benefits for hospital treatment and maybe even introducing catastrophic risk coverage for poor population and b) introducing or expanding drug benefits for chronic patients most importantly from lower income families. The current administrative system for social assistance allows delivering such increased benefits to poor. These policy recommendations may carry certain relevance for policy makers from other low and middle income countries facing similar problems of high OOP and catastrophic health expenditures.

## Conclusion

Reducing the prevalence of catastrophic health expenditure is a policy objective of the government, which can be achieved by focusing on increased financial protection offered to poor and expanding government financed benefits for poor and chronically ill by including and expanding inpatient coverage and adding drug benefits. These policy recommendations may also be relevant for other Low and Middle Income countries with similar levels of out of pocket payments and catastrophic health expenditures.

## Abbreviations

FFC: Fairness in Financial Contribution; GNI: Gross National Income; THE: Total Health Expenditure; HUES: Health Utilization and Expenditure Survey; HIS: Integrated Household Survey; WHO: World Health Organization; Gel: Georgian Lari (Local currency), OOP: Out-of-pocket Payment'; CTP: Capacity to Pay; HH: Household.

## Competing interests

The authors declare that they have no competing interests.

## Authors' contributions

GG conducted statistical analysis and drafted the manuscript. NR worked on the database (consistency checks etc.), conducted literature review, managed the fieldwork (piloting survey instrument, survey administration/interviewing process etc), and contributed to the data analysis. AZ reviewed the draft manuscript and compiled the final version. All authors have read and approved the final manuscript.

## Pre-publication history

The pre-publication history for this paper can be accessed here:


